# Computed Tomography Angiography Successfully Used to Diagnose Postoperative Systemic-Pulmonary Artery Shunt Narrowing

**DOI:** 10.1155/2011/802643

**Published:** 2011-07-20

**Authors:** Kurt D. Piggott, David G. Nykanen, Susan Smith

**Affiliations:** ^1^Arnold Palmer Hospital for Children, Congenital Heart Institute, Orlando, FL 32806, USA; ^2^Department of Pediatric Radiology, Arnold Palmer Hospital for Children, Orlando, FL 32806, USA

## Abstract

In recent years, there has been a marked reduction in surgical mortality for many complex forms of congenital heart disease. Treatment or palliative strategies vary but may include systemic-pulmonary central or Blalock-Taussig shunt. These shunts can be complicated by overcirculation, infection, thrombosis, and thromboembolism. Many diagnostic modalities are available to aide in diagnosis of postoperative shunt complications including echocardiography and cardiac catheterization but these may be invasive, inconclusive, or difficult to obtain adequate images. Computed tomography angiography (CTA) has many attributes that make it potentially useful in the evaluation of congenital heart disease and postoperative shunt complications. We report one patient where CTA guided the post-operative algorithm and appropriately identified a shunt narrowing despite repeated echocardiograms showing a patent shunt. These findings along with clinical suspicion appropriately guided us toward cardiac catheterization. To our knowledge, this is the first paper where CTA appropriately suspected a shunt narrowing in the absence of echocardiographic confirmation.

## 1. Introduction

In recent years, there has been a marked reduction in surgical mortality for many complex forms of congenital heart disease. Surgical strategies for many of these patients may include systemic-pulmonary central or Blalock-Taussig shunt and shunts can be complicated by overcirculation, infection, thrombosis, and thromboembolism. The diagnosis of these complications in the postoperative period can be challenging, and it is necessary to support clinical suspicion with diagnostic imaging to determine if additional intervention is necessary. Some of these modalities are less invasive but user dependent which can make them unreliable, while others are invasive and may be unnecessary.

Computed tomography angiography has been increasingly utilized to identify detailed cardiovascular anatomy both pre-and postoperatively [1–3]. It can be particularly useful in identifying pulmonary and systemic vascular structures that are difficult to visualize on echocardiography. The use of computed tomography angiography in evaluating intracardiac anomalies has been limited but is now changing with advances in multidetector scanners and gating techniques and should be considered as a means of postoperative or postcatheterization evaluation of shunts and stents as it is noninvasive and can be performed rapidly [1–3]. We believe that it may be particularly useful for postoperative patients where there is clinical suspicion for shunt stenosis.

We report one patient where computed tomography angiography guided the postoperative algorithm and appropriately diagnosed a shunt narrowing in the absence of echocardiographic confirmation. These findings appropriately determined whether or not cardiac catheterization was necessary with respect to the need for more invasive imaging.

## 2. Clinical Summary

**Figure 1:**
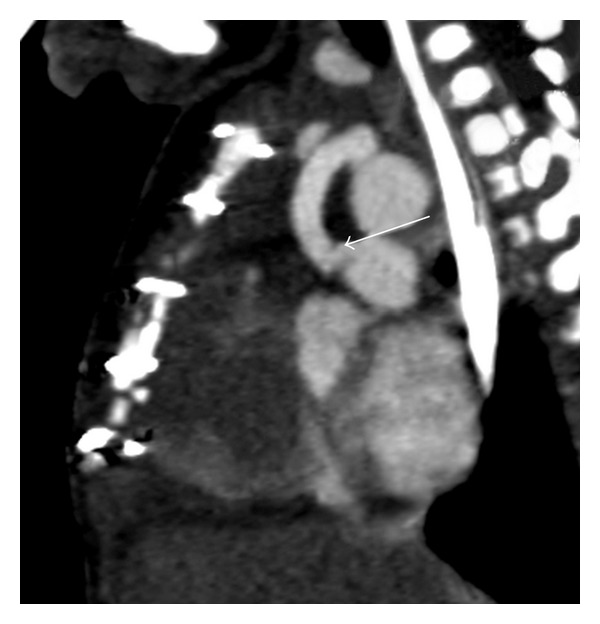
CT angiography (sagittal view) demonstrating a narrowing of the modified BT shunt at the point of anastamosis with the pulmonary artery.

**Figure 2:**
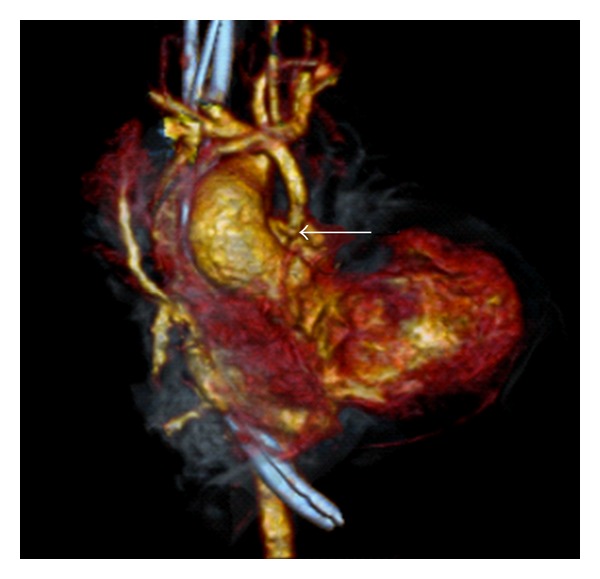
CT angiography with 3-dimensional reconstruction demonstrating a narrowing of the modified BT shunt at the point of anastamosis with the pulmonary artery.

**Figure 3:**
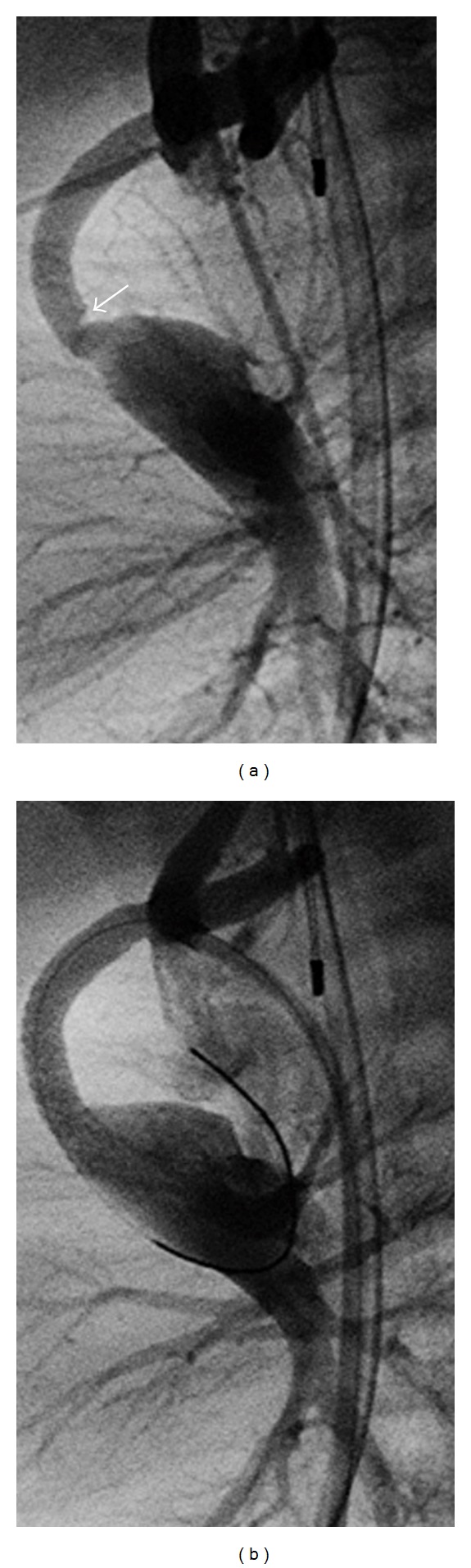
(a) angiographic (lateral) view during cardiac catheterization demonstrating shunt narrowing. (b) angiographic (lateral) view during cardiac catheterization demonstrating shunt poststent placement.

A 2-month-old female with tricuspid atresia, intact ventricular septum, absent pulmonary valve, and aneurysmal right ventricle. The subject of this paper is a 2 month old female. On day 4 of life, she underwent surgical palliation including placement of a 3.5 millimeter modified Blalock-Taussig shunt with transection and oversewing of the main pulmonary artery. Her initial postoperative course was uneventful. She had a normal blood pressure, normal heart rate, an audible shunt murmur, and oxygen saturation in room air of 75–85%. Mechanical ventilation was weaned, and on postoperative day 5 she was extubated. She failed extubation immediately requiring reintubation secondary to extrathoracic airway obstruction. The patient's ventilator was weaned a second time over a 5-day period. During this time period, she had 3 episodes of hypoxia requiring upward titration of ventilator settings. 2 of the episodes were transient and self-resolving. Chest radiographs were unremarkable and echocardiogram on both occasions showed shunt flow with no identified area of stenosis. On postoperative day 9 she had an episode of persistent hypoxia (oxygen saturation 50–60%) for 2 hours. A shunt murmur was noted, however, slightly diminished. The patient was started on a heparin infusion for presumed shunt thrombosis, and an echocardiogram was performed. Echocardiogram at that time showed adequate shunt flow and did not identify an area of narrowing. Repeated attempts to wean mechanical ventilation resulted in respiratory distress. On postoperative day 13, a computed tomography angiogram was performed which showed a stenosis of the shunt at the anastamosis of the right pulmonary artery (Figures 1 and 2). At cardiac catheterization, stenosis was confirmed and treated with stent implantation (Figures 3(a) and 3(b)). Over the next 2 days, her ventilator was weaned and she was extubated without complication.

## 3. Discussion

Diagnosing postoperative complications following surgery for congenital heart disease can be challenging in the setting of complex physiology. The diagnosis of shunt narrowing requires clinical suspicion and diagnostic imaging. Some imaging modalities are noninvasive but can be user dependent and technically limited.

Shunt thrombosis may be suggested on chest radiography as pulmonary oligemia, however, it is unreliable as the sole diagnostic modality. Echocardiography, while non-invasive and still considered the initial modality of choice in the workup of shunt narrowing, is user dependent and can be very challenging in the postoperative patient. In addition, it can be difficult to demonstrate early, partial occlusion, or shunt narrowing because flow will still be present. Cardiac catheterization is correctly the gold standard imaging technique, however, it is invasive and should be avoided if possible, particularly in the infant where future vascular access must be preserved.

Computed tomography angiography is increasingly useful in the evaluation of congenital heart disease, and it can also be employed to identify a variety of posttreatment complications [1–5]. Computed tomography angiography facilitates the assessment of extracardiac systemic and pulmonary arterial and venous structures. The use of computed tomography angiography in the role of intracardiac anomalies has been limited but is now changing with advances in multidetector scanners and gating techniques [1–5]. As seen in our patient, computed tomography angiography can be used to evaluate surgical shunt anatomy. Computed tomography angiography may be superior to magnetic resonance imaging for evaluation of stents because there is less metal-related artifact [1]. However, CTA can be problematic in the evaluation of stent patency when the stents are less than 5 millimeters. We believe that computed tomography angiography is a useful adjunct to echocardiography for evaluation of possible shunt narrowing or thrombosis, particularly in the setting of limited acoustic windows. Pulmonary artery anatomy may be difficult to visualize with echocardiography, while CT angiography is excellent at imaging pulmonary artery anatomy, and since the shunts are often anastomosed to these structures, it seems logical that it would be adequate, if not superior at identifying the entire length of the shunt. In addition, it is relatively fast and often requires little or no sedation. Conventional cardiac angiography may be limited by its 2-dimensional nature and because of its invasiveness, caries a higher rate of complication [6]. 

Computed tomography angiography is not the diagnostic modality of choice for all patients with suspected shunt narrowing. However, it should be considered in hemodynamically stable patients where there is a clinical suspicion for shunt narrowing which is otherwise unconfirmed by echocardiography. It can also guide imaging and transcatheter intervention at cardiac catheterization. 

Computed tomography angiography has been limited in this setting because of concerns of radiation exposure and limited resolution. With the development of new multi-slice scanners, radiation exposure is considerably less, resolution is much improved and acquisition is rapid [7, 8]. These features make it extremely attractive for use in children with congenital heart disease, and it should be considered in the postoperative period as a reliable and non-invasive method for evaluating systemic-pulmonary shunts.

We feel that our particular patient is worth reporting because CTA identified a discrete narrowing which confirmed our subtle clinical suspicion despite several echocardiograms failing to identify a shunt narrowing. We feel that early detection by CTA allowed the patient to undergo cardiac catheterization in a stable condition. If the narrowing had been allowed to progress, the patient would then require emergent catheterization and could suffer possible morbidities associated with this critical setting. While CTA is not a new modality in the evaluation of congenital heart disease, I believe its use in this particular setting is unique and worth reporting.

## 4. Conclusion

The rapid evolution of computed tomography angiographic technologies has rendered it an important diagnostic modality for patients with congenital heart disease. It may be applied in the postoperative patient where clinical suspicion is not corroborated by other diagnostic modalities and may obviate the need for more invasive cardiac catheterization.
